# Efficacy of Abdominal Acupuncture in Poststroke Constipation: A Systematic Review and Meta‐Analysis With Trial Sequential Analysis

**DOI:** 10.1002/brb3.71442

**Published:** 2026-04-29

**Authors:** Wulam Rukye, Hongzhi Yang, Yingjia Li, Xin Li, Nenggui Xu, Zhennan Wu

**Affiliations:** ^1^ South China Research Center for Acupuncture and Moxibustion, Medical College of Acu‐Moxi and Rehabilitation Guangzhou University of Chinese Medicine Guangzhou China; ^2^ The First College for Clinical Medicine Guangzhou University of Chinese Medicine Guangzhou China; ^3^ Department of Physiology University of Auckland Auckland New Zealand

**Keywords:** acupuncture, constipation, meta‐analysis, stroke

## Abstract

**Background:**

Poststroke constipation (PSC) is a prevalent and disabling condition that significantly affects patient quality of life. Although abdominal acupuncture (AA) has demonstrated potential therapeutic effects on PSC, its efficacy remains unclear. This meta‐analysis evaluated the efficacy of manual AA (MAA) and abdominal electroacupuncture (AEA) combined with conventional therapy (CT) for PSC.

**Methods:**

A comprehensive literature search was conducted to identify randomized controlled trials comparing the effects of MAA + CT or AEA + CT versus CT alone in PSC treatment. Twelve eligible studies (*n* = 943) were selected. Primary (efficacy and time to first bowel movement [BM]) and secondary (stool shape) outcomes were evaluated. Trial sequential analysis (TSA) was performed to assess cumulative evidence reliability.

**Results:**

Both MAA + CT (relative risk [RR] = 1.26, 95% confidence interval [CI]: 1.13–1.40) and AEA + CT (RR = 1.28, 95% CI: 1.19–1.38) significantly improved efficacy compared with CT alone. Furthermore, MAA + CT (mean difference [MD] = −0.61, 95% CI: −1.02 to −0.19) and AEA + CT (MD = −0.32, 95% CI: −1.09 to −0.46) reduced the time to first BM, albeit evidence regarding stool shape was inconclusive due to significant heterogeneity. TSA demonstrated unclear cumulative evidence and thus could not determine AA efficacy compared with CT.

**Conclusion:**

MAA + CT and AEA + CT may effectively improve PSC by enhancing efficacy and shortening the time to first BM. Given the limited number of studies, small sample sizes, and significant heterogeneity, further high‐quality trials are required to confirm these findings and optimize the acupuncture regimen for PSC treatment.

**Abbreviations**
AA: abdominal acupuncture
AEA: abdominal electroacupuncture
AEs: adverse effects
BM: bowel movement
CIs: confidence intervals
CSBM: complete spontaneous bowel movement
CT: conventional therapy
ENS: enteric nervous system
MAA: manual abdominal acupuncture
MD: mean difference
PRISMA: Preferred Reporting Items for Systematic Reviews and Meta‐Analyses
PSC: poststroke constipation
RCTs: randomized controlled trials
RDT: routine drug treatment
RIS: required information size
RR: relative risk
SMD: standardized mean difference
TCM: traditional Chinese medicine
TSA: trial sequential analysis
TSMB: trial sequential monitoring boundaries

## 1 Introduction

Poststroke constipation (PSC) is a frequent complication in patients with stroke, with prevalence rates exceeding 50% among stroke survivors (Yong et al. 2023). PSC symptoms include difficulty in defecation, abdominal distension, a sensation of incomplete evacuation, and abdominal discomfort. In severe cases, constipation can lead to bowel obstruction and intestinal ischemia. PSC significantly affects patients’ physical health and quality of life. PSC symptoms, including abdominal bloating, pain, and defecation difficulties, can trigger a decline in self‐care ability, reduced independence, and an increased psychological burden (Fan et al. 2019). This may have a detrimental effect on mental health, potentially causing depression and anxiety. Moreover, constipation is correlated with an elevated risk of recurrent stroke. Increased abdominal pressure induced by constipation precipitates fluctuations in blood pressure, engendering cerebral blood flow instability. This phenomenon is of particular concern in patients with stroke, as constipation can exacerbate hemodynamic instability, thereby increasing the risk of recurrent stroke with potentially fatal consequences (Lin et al. 2013).

PSC occurrence is associated with many factors (Su et al. 2009). First, stroke can damage the autonomic nervous system, thereby affecting neural intestinal regulation and triggering peristaltic and defecation reflex impairment. Second, stroke‐induced motor impairments reduce physical activity and slow intestinal motility. Constipation is further compounded by insufficient fiber and fluid intake. Moreover, patients with stroke lesions in the right frontal gyrus, left superior parietal lobule, bilateral precentral gyrus, postcentral gyrus, insula, and inferior parietal lobule should be particularly vigilant regarding constipation (Han et al. 2023).

The current corpus of clinical treatment strategies is based on clinical practice guidelines for functional constipation (Chang et al. 2022). The most commonly prescribed medications include laxatives, stool softeners, and cathartics. However, these pharmaceutical agents are frequently associated with adverse effects (AEs), including diarrhea, electrolyte imbalance, and dependency, particularly with prolonged use, which may impede normal bowel function. Furthermore, the use of antidepressants and diuretics exacerbates constipation. Research has indicated that constipation may be indicative of cardiovascular risk factor exposure, whereas the use of laxatives potentially increases the risk of ischemic stroke and mortality from coronary heart disease (Kubota et al. 2016). Consequently, the efficacy of pharmacological treatments is limited, and the associated side effects pose a significant risk to patients undergoing long‐term therapy. This underscores the pressing need to explore more effective and safer treatment modalities.

Several meta‐analyses have comprehensively evaluated the efficacy of acupuncture in patients with PSC (Sun et al. 2023; Yang et al. 2014; Tang et al. 2020). Acupuncture elicited curative effects with constipation improvement in these patients. However, most of these studies focused on evaluating the efficacy of traditional acupuncture treatment, which mainly achieves therapeutic outcomes via whole‐body acupoint stimulation. Moreover, whole‐body acupoint stimulation encompasses a broader acupoint selection and meridian regulation, emphasizing systemic physiological balance.

Abdominal acupuncture (AA) is a traditional Chinese medicine (TCM) treatment involving acupuncture at specific points on the abdomen. Its theoretical basis comes from the “abdominal‐brain theory” and the theory that “the stomach gate communicates with the five zang organs and six fu organs” of TCM. Recent studies have shown that AA is effective in PSC treatment (Su et al. 2021; Zhan et al. 2021).

Stimulating specific acupoints that are closely related to gastrointestinal function can directly regulate motor function and blood circulation in the gastrointestinal tract, thereby relieving gastrointestinal symptoms (Yi et al. 2021; Wang 2022; Ding et al. 2024).

Given the potential advantages of AA in PSC treatment (Jiang et al. 2024; Liu et al. 2023; Chen and Erdunf 2022; Wu 2019; Sun and Song 2019; Peng 2019; Li et al. 2019; Xiong et al. 2017; Xie et al. 2016; Zhang 2015; Zeng et al. 2013; Wang et al. 2008), and the paucity of systematic literature reviews on this subject, this systematic review and meta‐analysis aimed to comprehensively evaluate the efficacy of AA through combined trial sequential analysis (TSA) and further explore the advantages and potential mechanisms of AA. Through this study, we aim to provide evidence for clinical treatment as well as a theoretical basis and practical guidance for the application of AA in the treatment of constipation associated with neurological diseases.

## 2 Methods

### 2.1 Study Registration

The current study was conducted in accordance with the Preferred Reporting Items for Systematic Reviews and Meta‐Analyses (PRISMA) guidelines (Page et al. 2021). The study was prospectively registered in the International Prospective Register of Systematic Reviews on December 7, 2024 (Registration number CRD42024619280).

### 2.2 Search Strategy

A systematic search protocol was implemented across four major English‐language biomedical databases (Cochrane Library, PubMed/MEDLINE, Embase, and Web of Science Core Collection) and four Chinese academic repositories (China Biology Medicine disc, VIP Chinese Journal Database, Wanfang Med Online, and CNKI Scholar), with search parameters constrained to studies published before January 31, 2025 (Supporting Information File 1).

### 2.3 Inclusion Criteria

#### 2.3.1 Study Design

Randomized controlled trials (RCTs) of AA in PSC.

#### 2.3.2 Patients

The study participants were patients diagnosed with PSC. Constipation was diagnosed according to the Rome II, III, and IV criteria (Drossman 1999, 2006, 2016) or the Chinese Medical Association Provisional Standards for the Diagnosis and Treatment of Constipation (Chinese Medical Association SB, Coloproctology Group 2000), Guiding Principles for Clinical Research of New Chinese Medicines (2002) (Zheng 2002), and State Administration of TCM (Medicine SAoTC 1996). Stroke was diagnosed according to the Chinese Guide for Diagnosis and Treatment of Acute Ischemic Stroke or based on magnetic resonance imaging or computed tomographic findings.

#### 2.3.3 Intervention

The experimental group received AA as monotherapy or as an adjuvant to conventional therapy (CT), which included manual AA (MAA) and abdominal electroacupuncture (AEA).

#### 2.3.4 Comparison

The participants in the control group were administered either sham acupuncture or CT. CTs were limited to fibers, osmotic laxatives, stimulant laxatives, and gastrointestinal prokinetics. No restrictions were imposed on dosage, route of administration, or duration of treatment.

#### 2.3.5 Outcomes

The trials included in this analysis reported at least one primary outcome, which was defined as either the total response rate or the constipation symptom score. Secondary outcomes included AEs and time to first bowel movement (BM). The total response rate was defined as the proportion of patients who demonstrated improvement in their symptoms. The constipation symptom score was based on the constipation symptom scale developed according to the Chinese consensus on the diagnosis and treatment of chronic constipation.

### 2.4 Exclusion Criteria

We excluded (Yong et al. 2023) other trials of Chinese medicine treatments, such as Chinese herbal medicine, massage, acupuncture, auricular acupuncture, cupping and embedding thread therapy, and comparative trials of different acupuncture methods (Fan et al. 2019); trials with incomplete or incorrect data; and (Lin et al. 2013) trials for which the full text was not available.

### 2.5 Data Extraction

Following the conclusion of the search, the retrieved literature was imported into EndNote X9 software. Two reviewers (Wulam Rukye and Hongzhi Yang) then independently screened the titles and abstracts of 556 articles, excluding 328 studies that did not meet the inclusion criteria. The remaining studies were reviewed in full text to determine their relevance to the inclusion criteria. The data extraction process was conducted by two review authors and encompassed details, such as the year of publication, the lead author, the randomization technique, the sample size, the grouping, the age, gender, and disease duration of the participants, the intervention, including the number, location, and setting of the acupuncture points, the outcome data, and any AEs. In the event of disagreement, a third reviewer (Zhennan Wu) was consulted to reach a final decision.

### 2.6 Assessment of Quality

The potential for bias in the literature was evaluated using Cochrane RoB 2.0 (Sterne et al. 2019). Each RCT was assessed against six criteria as follows: randomization process, unintended interventions, missing outcome data, measurement of results, reporting of results, and overall assessment. The study was considered to have a low risk of bias if the research methods were deemed appropriate and adequately explained and if the study was reported to be of high quality. Conversely, if the research methods were unclear or problematic, the study was considered to have a high risk of bias. Two researchers (Yingjia Li and Hongzhi) Yang independently assessed these factors, and any disagreements were resolved by consultation with a third researcher (Xin Li).

### 2.7 Statistical Analysis

Data analysis was applied using the following process (Wu et al. 2024). The “metafor” package in *R* was used to statistically evaluate the included RCTs. Effect sizes were determined by analyzing posttreatment differences between groups. Continuous variables were reported as mean difference (MD) or standardized MD (SMD) with 95% confidence intervals (CIs). Study heterogeneity was assessed using *I* ^2^ and *p* values, with *I* ^2^ values > 50% or *p* values < 0.01 indicating significant heterogeneity. In instances where heterogeneity was identified, a random‐effects model was employed; otherwise, a fixed‐effects model was used.

Subgroup analyses were conducted to investigate potential sources of heterogeneity, and sensitivity analyses were performed using the exclusion method to verify the robustness of the results. Furthermore, TSA was employed to ensure the reliability of the research findings. TSA integrates interim analyses of RCTs and meta‐analysis to provide a more robust statistical framework.

In contrast to conventional meta‐analyses, TSA effectively controls for random and Type I errors (false positives) by determining the required information size (RIS) and establishing trial sequential monitoring boundaries (TSMB). The analysis was performed using TSA software version 0.9.5.10 beta (https://www.ctu.dk/tsa/). TSA calculates both the RIS and TSMB and assesses the sufficiency of existing evidence based on cumulative sample size at a defined *α* (0.05) and *β* (0.2) level. When the cumulative sample size reaches or exceeds the RIS, the evidence is considered adequate to derive reliable conclusions. When this threshold is not met, additional high‐quality studies are required to confirm the findings.

TSA was used to minimize random errors arising from repeated analyses and ensure the statistical validity of the conclusions. The implementation of a boundary value and subsequent RIS determination simplified the evaluation of evidence sufficiency and robustness, thereby enhancing the credibility of the study findings.

## 3 Results

### 3.1 Study Selection

**FIGURE 1:**
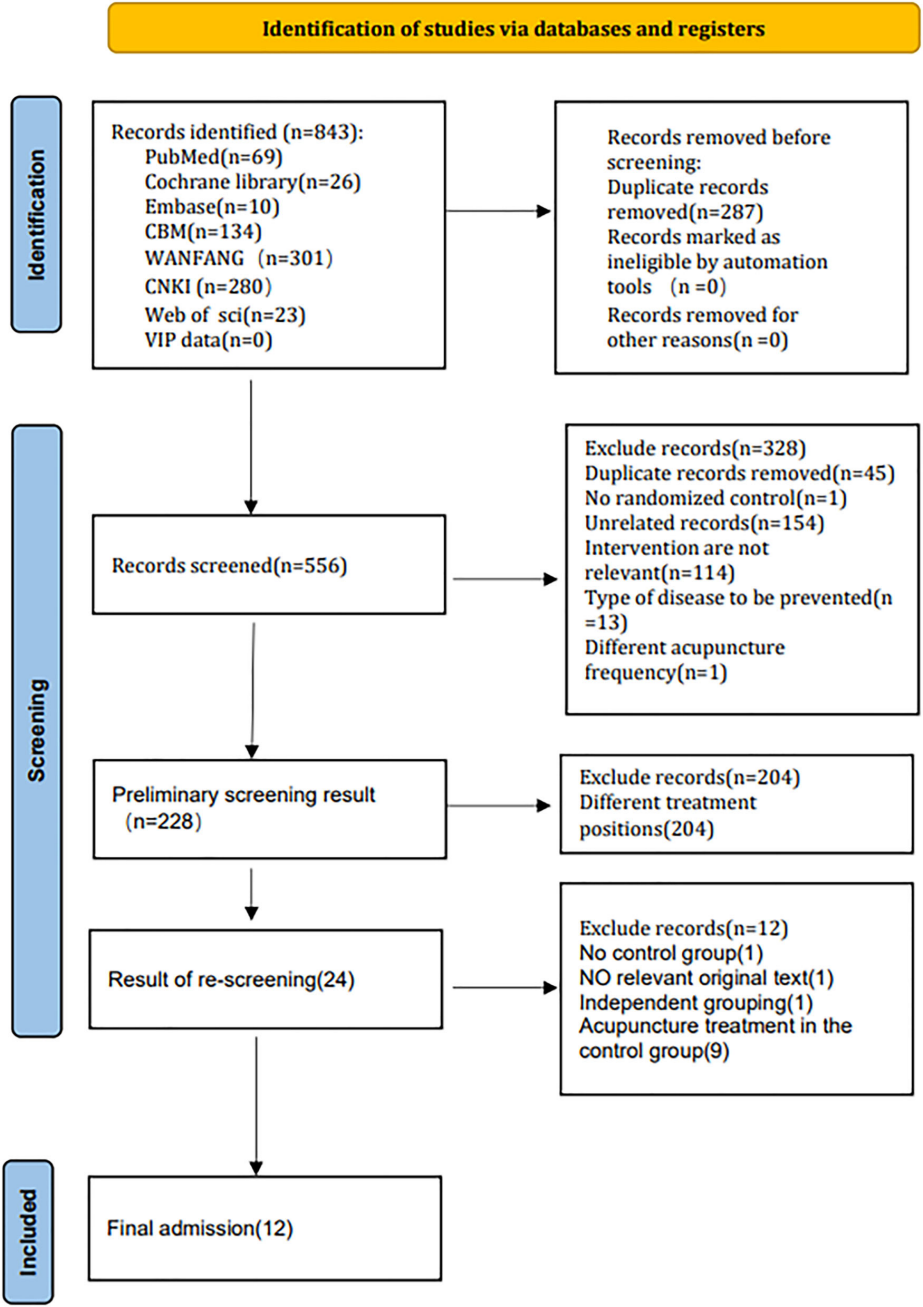
PRISMA flow diagram of the study selection process. The flowchart details the identification, screening, and inclusion of records from various databases. It outlines the number of studies excluded at each stage (e.g., duplicate records and irrelevant interventions) and the reasons for exclusion, resulting in the final 12 RCTs included in the systematic review and meta‐analysis.

Figure 1 shows the PRISMA flow diagram for the literature selection. In total, 843 records were retrieved from the databases. Before the screening, 287 duplicate records were removed. After screening titles and abstracts, we excluded 532 articles on the basis of the inclusion criteria and selected 24 articles for full‐text review. Finally, 12 articles were included in this meta‐analysis (Table 1). Regarding the diagnosis of constipation, eight, one, two, and one study used the Rome criteria (Jiang et al. 2024; Liu et al. 2023; Chen and Erdunf 2022; Wu 2019; Sun and Song 2019; Peng 2019; Li et al. 2019; Xiong et al. 2017), Chinese New Clinical Criteria for TCM (Zhang 2015), Provisional Standards for the Diagnosis and Treatment of Constipation (Zeng et al. 2013; Wang et al. 2008), and State Administration of TCM, respectively (Xie et al. 2016). The criteria for stroke in all included articles referred to computed tomography‐ or magnetic resonance imaging‐confirmed cerebral infarction or cerebral hemorrhage. One study was reported in English (Liu et al. 2023), and the rest were in Chinese.

**TABLE 1:** Characteristic of the include studies.

References	Digotise	Randomization	Sample size	Average age (mean ± SD)	IG	CG	Intervention	Duration (week)	Frequency	Acupoints	Outcomes
G. F. Peng (2019)	①④	PG	IG = 33 CG = 33	IG = 59.8 ± 9.41 CG = 60.2 ± 9.23	MAA + CT	CT	MAA	2	QD, STW	CV12, CV10, CV6, CV4, ST25, SP15, LV2	⑥
Z. H. Xiong et al. (2017)	①④	PG	IG = 80 CG = 80	IG = 60 ± 5 CG = 60 ± 5	AEA	CT	AEA	10 days	QD	ST25, SP14, SP15, ST29, CV6, CV4, CV8	⑦⑧
G. P. Liu et al. (2023)	①④	RML	IG = 46 CG = 46	IG = 60.2 ± 9.1 CG = 60.2 ± 8.8	AEA + CT	CT	AEA	2	THW	ST25, SP13, SP15	⑦⑧
D. S. Wang et al. (2008)	②④	PG	IG = 40 CG = 40	IG = 63.2 ± 3.74 CG = 61.9 ± 4.65	AEA	CT	AEA	2	THW	SP15, SP14, ST25, ST28	⑥⑧
H. M. Zhang (2015)	③④	PG	IG = 30 CG = 30	IG = 59.631 ± 7.91 CG = 59.33 ± 11.604	MAA	RM	MAA	2	ALT	LI11, HT1, GB34, SP6, LR3, SP14	⑦⑧
Z. P. Zeng (2013)	②④	PG	IG = 40 CG = 40	/	MAA + CT	CT	MAA	1	QD	CV12, ST25, SP15, ST28, ST29, ST36	⑧
S. C. Li (2019)	①④	RML	IG = 40 CG = 40	IG = 62.7 ± 8.5 CG = 62.5 ± 8.6	MAA + CT	CT	MAA	4	QD	CV12, ST25, SP15, CV6, CV4	⑥
L. X. Sun and Song (2019)	①④	RML	IG = 43 CG = 43	IG = 61.46 ± 7.03 CG = 61.68 ± 7.24	MAA + CT	CT	MAA	2	QD	CV12, CV10, CV6, CV4, ST25, SP15	⑦⑧
L. Wu (2019)	①④	RML	IG = 23 CG = 23	IG = 62.26 ± 8.48 CG = 61.60 ± 7.75	MAA + CT	CT	MAA	12 days	QD	ST25, ST24, ST26, CV12, CV10, CV4, CV6, EX‐UE9, EX‐LE10	⑧
G. J. Chen (2022)	①④	PG	IG = 30 CG = 30	/	MAA	CT	MAA	2	QD	Abdominal acupuncture	⑧
Y. W. Jiang et al. (2024)	①④	PG	IG = 36 CG = 37	IG = 66. 33 ± 5. 88 CG = 66. 32 ± 6. 41	AEA	CT	AEA	4	QD, FTW	ST25, CV6, CV4	⑦
H. Xie et al. (2016)	④⑤	PG	IG = 30 CG = 30	IG = 65.40 ± 9.89 CG = 60.50 ± 10.47	MAA + CT	CT	MAA	2	QD	ST25, EX‐LE4, ST36, BL25, LU6	⑧

*Note*: ① International Functional Gastrointestinal Disorders (FGIDs)—RomanII/III/IV Diagnostic Criteria. ② Chinese Medical Association ‘Provisional Standards for the Diagnosis and Treatment of Constipation (1999). ③ Guiding Principles for Clinical Research of New Chinese Medicines (2002). ④ CT/MRI confirmed cerebral infarction or cerebral hemorrhage. ⑤ Chinese Medicine Diagnostic Criteria for Constipation. ⑥ Symptom score. ⑦ Time of first defecation. ⑧ Total effective rate.

Abbreviations: AEA, abdominal electroacupuncture; ALT, alternate day; CG, control group; CT, conventional therapy; FTW, five times a week; IG, intervention group; MAA, manual abdominal acupuncture; PG, parity grouping; QD, one a day; RM, routine manage; RML, random number list; SANR, sun's abdominal nine regions; STW, six times a week; THW, three times a week.

### 3.2 Study Characteristics

#### 3.2.1 Patients

Table 1 gives a summary of the basic details of the studies that were included. All 12 articles were studies that were conducted in China between 2008 and 2024. One of the articles was published in English, and the rest were published in Chinese. In total, there were 943 patients who met the inclusion criteria, 471 of whom were in the experimental group and 472 of whom were in the control group.

#### 3.2.2 Acupuncture Interventions

In the included trials, acupuncture interventions mainly included MAA (Chen and Erdunf 2022; Wu 2019; Sun and Song 2019; Peng 2019; Li et al. 2019; Xie et al. 2016; Zhang 2015; Zeng et al. 2013) and AEA (Jiang et al. 2024; Liu et al. 2023; Xiong et al. 2017; Wang et al. 2008). Certain study groups received combined treatments, such as MAA or AEA combined with routine drug treatment (RDT) and MAA or AEA combined with TCM; one study group received MAA combined with auricular therapy. The duration of treatment varied between trials, ranging from 1 to 4 weeks, with a few trials providing treatment daily, three times a week, or on alternate days (Table 1).

#### 3.2.3 Control Measures

The control groups in the included studies primarily underwent conventional treatments or routine management.

### 3.3 Risk of Bias

**FIGURE 2:**
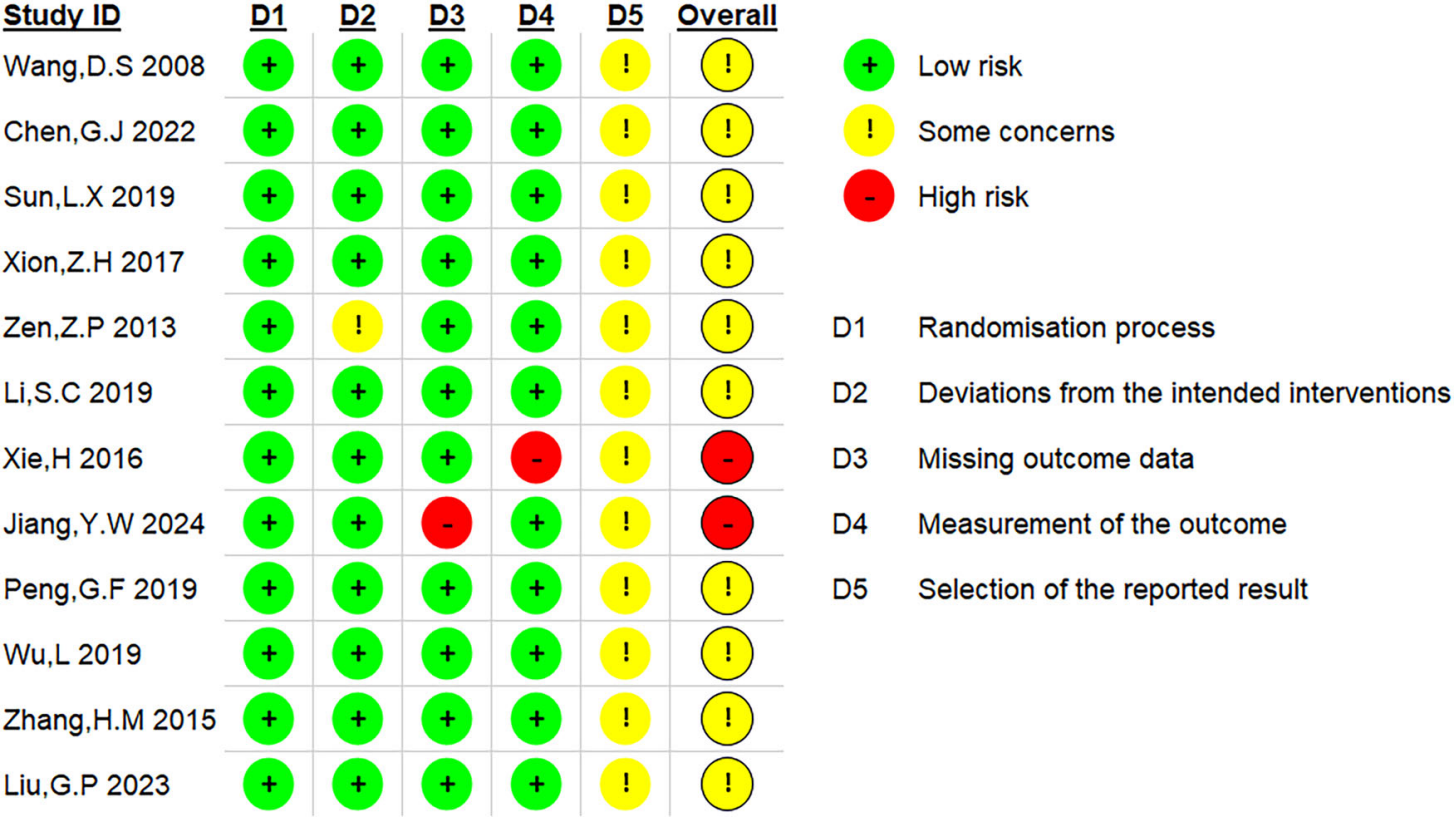
Risk of bias. The figure presents the risk of bias assessment for each included randomized controlled trial using the Cochrane RoB 2.0 tool. The assessment evaluates five distinct domains (D1: Randomization process; D2: Deviations from intended interventions; D3: Missing outcome data; D4: Measurement of the outcome; and D5: Selection of the reported result) and provides an overall risk of bias rating (low risk, some concerns, or high risk) for each study.

**FIGURE 3:**
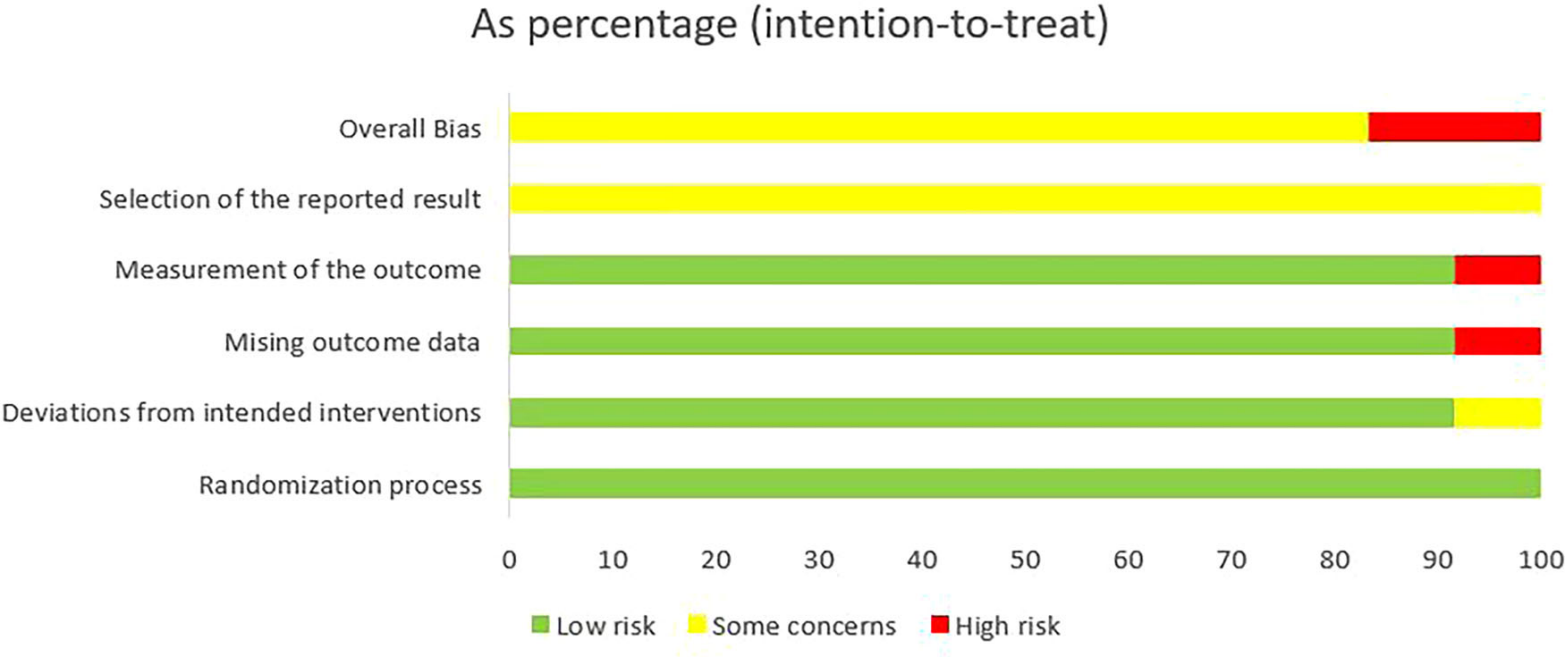
Summary of risk of bias. The graph illustrates the proportion of all included studies assigned to each risk category (low risk, some concerns, or high risk) across the specific Cochrane RoB 2.0 assessment domains. Data are presented as intention‐to‐treat percentages to visualize the overall methodological quality of the synthesized evidence.

Regarding the risk of bias analyses, 10 and 2 RCTs had moderate (Liu et al. 2023; Chen and Erdunf 2022; Wu 2019; Sun and Song 2019; Peng 2019; Li et al. 2019; Xiong et al. 2017; Zhang 2015; Zeng et al. 2013; Wang et al. 2008) and high risks of bias, respectively. Certain concerns exist regarding the selection of reported results in all articles. Of the two articles with a high risk of bias, bias resulted from missing outcome data in one (Xie et al. 2016) and outcome measurement in the other (Jiang et al. 2024) (Figures 2 and 3).

### 3.4 Primary Outcomes

#### 3.4.1 Efficacy Rate

MAA + CT and AEA + CT combinations significantly improved efficacy rates compared to CT alone. The relative risks (RRs) for MAA + CT and AEA + CT were 1.26 (95% CI: 1.13–1.40) and 1.28 (95% CI: 1.19–1.38), respectively. These results suggest that both forms of AA combined with CT were more effective than CT alone.

**FIGURE 4:**
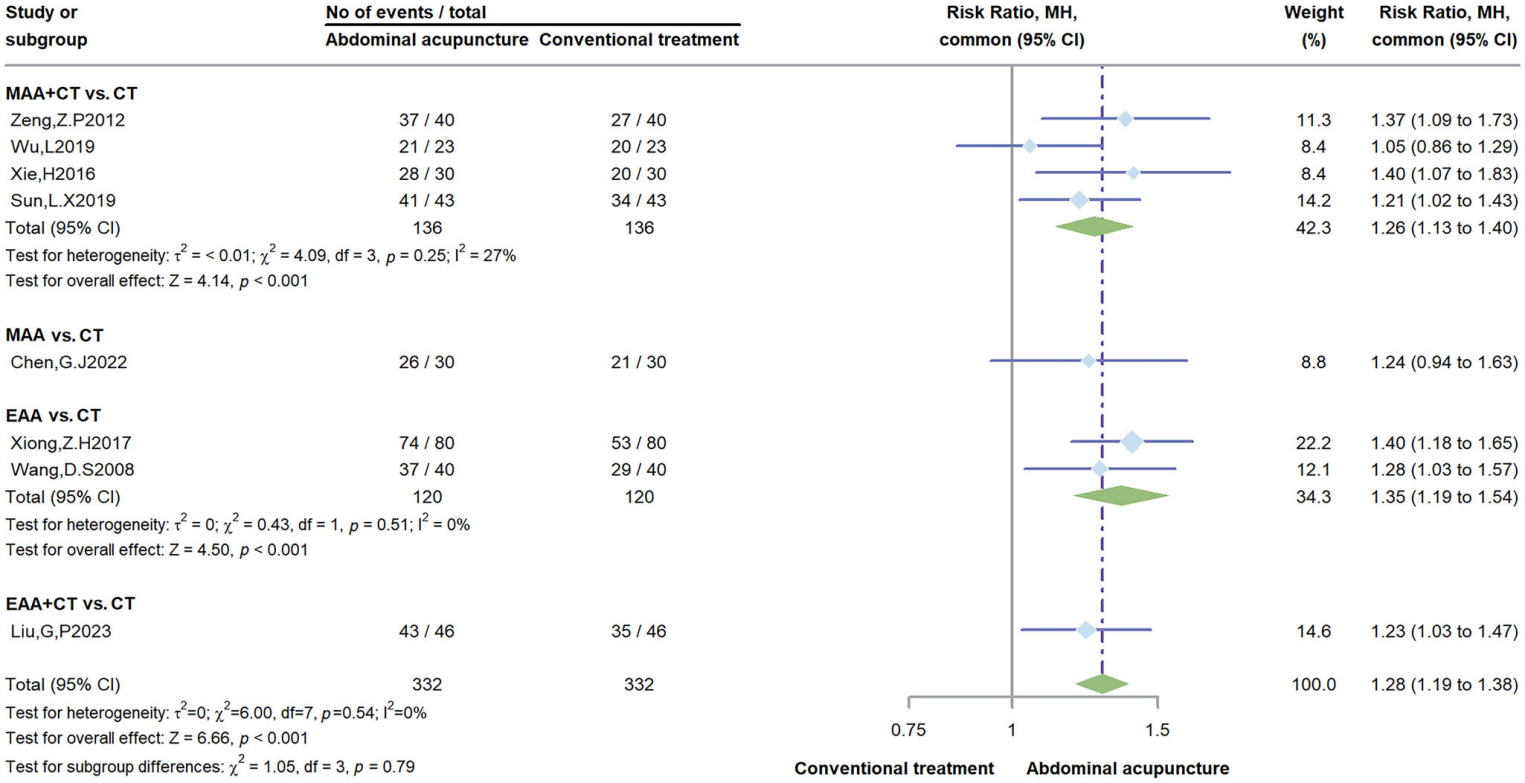
Forestplot of effective rate. The analysis compares different intervention subgroups as follows: MAA combined with CT, MAA alone, AEA alone, and AEA combined with CT, all compared against CT alone. Effect sizes are presented as RR with 95% CI using a common (fixed) effects model. The size of the data markers indicates the statistical weight of each study, and the diamonds represent the pooled overall effects for the subgroups and the total. CI, confidence interval; CT, conventional therapy; MAA, manual abdominal acupuncture.

In contrast, when MAA was assessed separately from CT, there was no significant difference in efficacy (RR = 1.24, 95% CI: 0.94–1.63). This indicates that the efficacy of MAA alone is unclear (Figure 4).

### 3.5 Secondary Outcomes

#### 3.5.1 Time to First Constipation Episode

Regarding the comparison of MAA + CT versus CT, a significant difference in the time to first constipation episode was observed, with an MD of −0.61 (95% CI: −1.02 to −0.19), suggesting that MAA + CT may result in faster constipation resolution.

**FIGURE 5:**
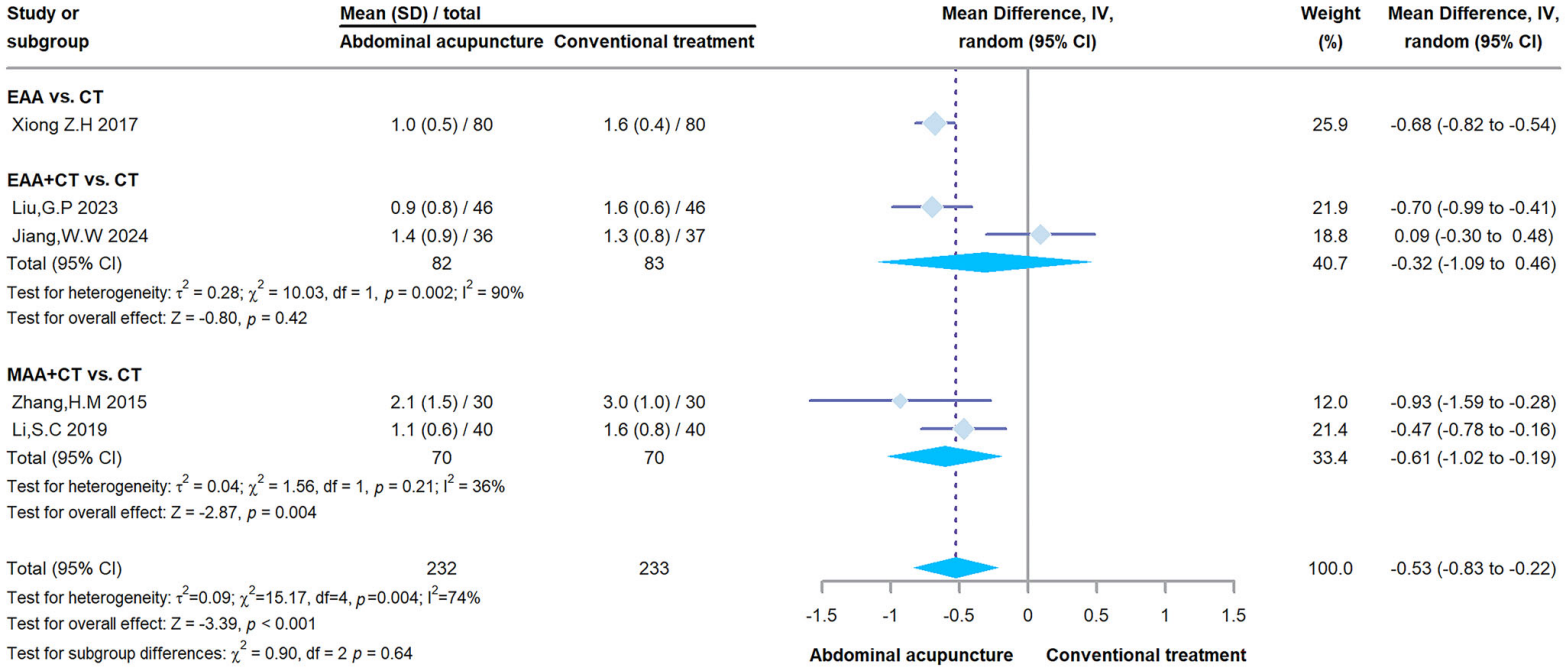
Forestplot of time to first constipation episode. The plot compares the efficacy of AEA versus CT, AEA + CT versus CT, and MAA + CT versus CT in reducing the time to the first BM. Continuous data are expressed as MD with 95% CI utilizing a random‐effects model. The plot details the sample sizes, mean values, and standard deviations for the experimental and control groups. CI, confidence interval; CT, conventional therapy; MAA, manual abdominal acupuncture.

Similarly, the comparison of AEA + CT versus CT showed a statistically significant difference (MD = −0.32, 95% CI: −1.09 to −0.46) (Figure 5).

#### 3.5.2 Stool Shape

**FIGURE 6:**
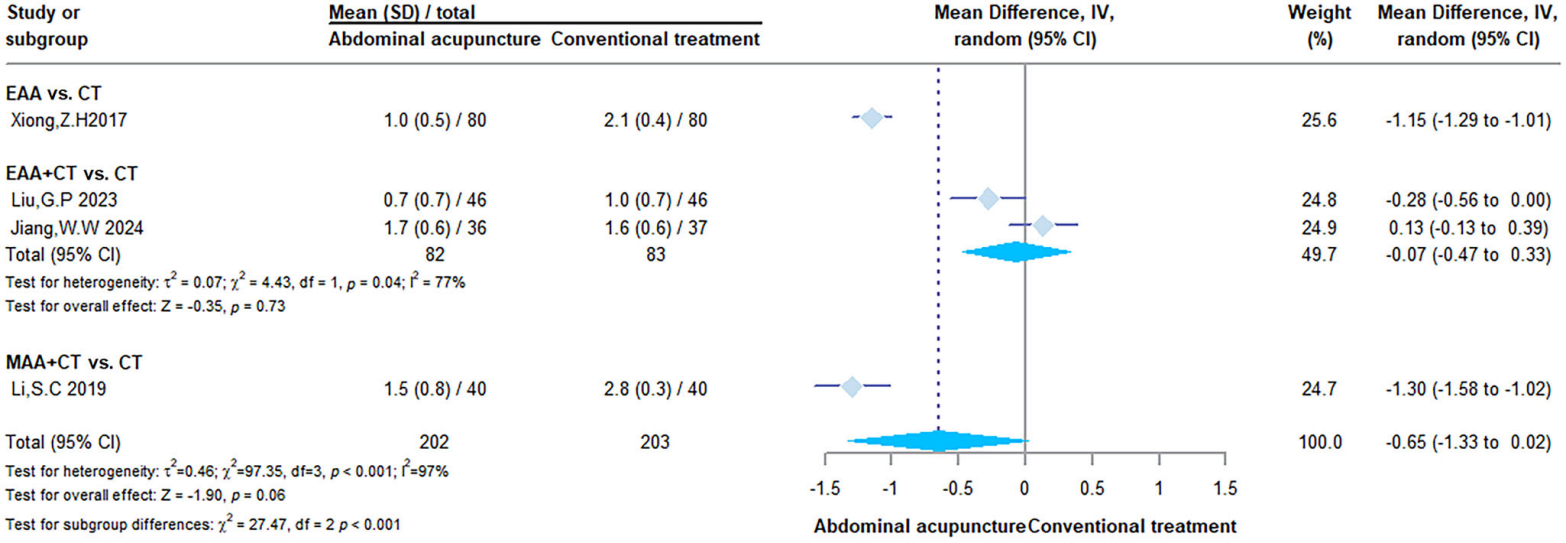
Forestplot of stool shape. The subgroups compare different AA modalities (AEA, AEA + CT, and MAA + CT) against CT for improving stool shape. Data are represented as MD with 95% CI under a random‐effects model, reflecting the substantial clinical and statistical heterogeneity present across the trials. CI, confidence interval; CT, conventional therapy; MAA, manual abdominal acupuncture.

The stool shape was evaluated in fewer studies, and the findings were inconsistent across studies. After sensitivity analysis (S2), for MAA + CT, the SMD was −0.18 (95% CI: −0.71 to 0.34); no significant difference was noted for AEA + CT (SMD = −0.01, 95% CI: −0.71 to 0.70). In addition, substantial heterogeneity was observed in both groups (*I* ^2^ = 81%), further weakening the evidence (Figure 6).

### 3.6 Trial Sequential Analysis

**FIGURE 7:**
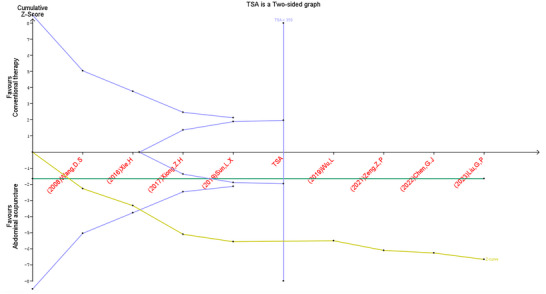
TSA of effective rate. The TSA evaluates the reliability of the cumulative evidence regarding the efficacy of abdominal acupuncture compared to conventional therapy. TSA, trial sequential analysis.

To assess the reliability of our findings and determine if the cumulative evidence for the efficacy rate was conclusive, we performed a TSA. TSA helps control for the risk of random errors (Type I errors) due to sparse data and repeated significance testing in cumulative meta‐analyses. We calculated the RIS on the basis of a Type I error (*α*) of 5% and a power (1 − *β*) of 80%. The TSA plot for the efficacy rate is shown in Figure 7. The analysis calculated a RIS of 350 participants needed to draw a firm conclusion. The cumulative *Z*‐curve (yellow line) represents the evidence accumulated with each successive trial. Our interpretation of the TSA reveals several key points as follows: (1) The cumulative *Z*‐curve consistently remained in the lower portion of the graph, indicating a persistent trend favoring AA over CT. Despite this trend, the *Z*‐curve failed to cross the trial sequential monitoring boundary for benefit (the lower sloping blue line). This means that, despite the positive trend, the cumulative evidence has not yet reached the threshold for statistical conclusiveness. Crucially, the total number of participants included in the meta‐analysis (*n* = 943) has already surpassed the calculated RIS of 350 participants (the vertical blue line). The fact that the cumulative sample size exceeds the RIS, yet the *Z*‐curve has not crossed a significance boundary, suggests that the true effect size is likely smaller than what was anticipated when calculating the RIS, or that heterogeneity among the studies has prevented the evidence from becoming definitive. Therefore, although the results are promising, the TSA demonstrates that the current evidence is not yet conclusive. More high‐quality, large‐scale RCTs are still needed to definitively confirm the efficacy of AA for PSC.

### 3.7 GRADE System

Table 2 provides the results of the GRADE system evaluation. For the specific reasons for downgrading, please refer to S3.

**TABLE 2:** Summary findings of meta‐analysis and evidence grade.

Outcome	Result	No. of studies (participants)	Grade of evidence	Favor
**MAA** + **CT** vs. **CT**				
Effective rate	*p* = 0.25, 𝐼^2^ = 27%, RR = 1.26, 95% CI (1.13–1.40), $X_{3}^{2}$ = 4.09	4 studies, 136 participants	Low‐quality^a^	Manual abdominal acupuncture + conventional treatment
Time to first constipation	*p* = 0.21, 𝐼^2^ = 36%, MD = −0.61, 95% CI (−1.02 to 0.19), $X_{1}^{2}$ = 1.56	2 studies, 70 participants	Moderate‐quality^b^	Manual abdominal acupuncture + conventional treatment
Shape of constipation	N/A, SMD = −0.18, 95% CI (−0.71 to 0.34)	1 studies, 40 participants	Very low‐quality^[Link]^, ^b^	Manual abdominal acupuncture + conventional treatment
**AEA** + **CT vs. CT**				
Effective rate	N/A, RR = 1.28, 95% CI (1.19–1.38)	1 studies, 46 participants	Moderate‐quality^a^	Electric abdominal acupuncture + conventional treatment
Time to first constipation	*p* = 0.002, 𝐼^2^ = 90%, MD = −0.32, 95% CI (−1.09 to 0.46), $X_{1}^{2}$ = 10.03	2 studies, 83 participants	Low‐quality^b^	Electric abdominal acupuncture + conventional treatment
Shape of constipation	*p* = 0.02, 𝐼^2^ = 81%, SMD = −0.01, 95% CI (−0.71 to 0.70), $X_{1}^{2}$ = 5.18	2 studies, 83 participants	Very low‐quality^[Link]^, ^b^	Electric abdominal acupuncture
**MAA vs. CT**				
Effective rate	N/A, RR = 1.24, 95% CI (0.94–1.63)	1 studies, 30 participants	Low‐quality^a^	Manual abdominal acupuncture
**AEA vs. CT**				
Effective rate	*p* = 0.51, 𝐼^2^ = 0%, RR = 1.35, 95% CI (1.19–1.54), $X_{1}^{2}$= 0.43	2 studies, 120 participants	Moderate‐quality^a^	Electric abdominal acupuncture
Time to first constipation	N/A, MD = −0.68, 95% CI (−0.82 to 0.54)	1 studies, 80 participants	Low‐quality^b^	Electric abdominal acupuncture

Abbreviations: AEA, abdominal electroacupuncture; CI, confidence interval; CT, conventional therapy; MAA, manual abdominal acupuncture; MD, mean difference; RR, relative risk; SMD, standardized mean difference.

^a^ Aricle had a wide confidence interval or a small simple size.

^b^ The heterogeneity of subgroup was relatively high.

#### 3.7.1 MAA + CT vs. CT

Efficacy (low quality): The RR was 1.26 (95% CI: 1.13–1.40), suggesting that MAA + CT may improve efficacy.

Time to first defecation (moderate quality): The MD was −0.61 (95% CI: −1.02 to −0.19), suggesting that MAA may have a role in promoting the time to first defecation.

Stool shape (very low quality): As only one study with a small sample size (*n* = 40) was included, the quality of evidence was very low, making it difficult to draw a clear conclusion.

#### 3.7.2 AEA + CT vs. CT

Efficacy (moderate quality): The RR was 1.28 (95% CI: 1.19–1.38), suggesting that AEA combined with routine treatment may be more effective than routine treatment alone.

Time to first defecation (low quality): The MD was −0.32 (95% CI: −1.09 to −0.46) and *I* ^2^ *=* 90%, suggesting that the intervention may be effective in shortening the time to first defecation, but the high heterogeneity reduced the quality of evidence.

Stool shape (very low‐quality evidence): The SMD was −0.01 (95% CI: −0.71 to 0.70). Only two studies were included, and there was high heterogeneity (*I* ^2^ = 81%), resulting in very low‐quality evidence.

#### 3.7.3 MAA vs. CT

Efficacy (low quality): The RR was 1.24 (95% CI: 0.94–1.63). Although this suggests a possible improvement, the small number of included studies and wide CIs affected the assessment of the quality of evidence.

#### 3.7.4 AEA vs. CT

Efficacy (moderate quality): The RR was 1.35 (95% CI: 1.19–1.54) and *I* ^2^ = 0%, suggesting that AEA may improve the efficacy of constipation treatment; moreover, the low heterogeneity supports its efficacy.

Time to first defecation (low quality): The MD was −0.68 (95% CI: −0.82 to −0.54); only one study was included, resulting in low‐quality evidence.

### 3.8 Adverse Effects

Although four articles (Liu et al. 2023; Wu 2019; Peng 2019; Zeng et al. 2013) mentioned the recording of AEs, none reported the occurrence of AEs, making it difficult to conclude about treatment safety.

## 4 Discussion

To fully appreciate the clinical value of AA for PSC, it is essential to understand its unique theoretical foundation, which distinguishes it from general body acupuncture. Developed by Professor Bo Zhiyun, AA is a specialized microsystem acupuncture technique based on the Shenque (CV8) meridian system. According to TCM theory, the abdomen is the center of the zang–fu organs and the root of Qi and blood. Although general acupuncture relies on the 14 major meridians distributed across the entire body, AA focuses exclusively on the abdominal region, utilizing acupoints primarily along the Ren and Kidney meridians (e.g., Zhongwan CV12, Tianshu ST25, and Guanyuan CV4). In the context of stroke, patients often suffer from Qi deficiency and blood stasis, leading to a failure in the transmission function of the large intestine. AA directly targets the core of the zang–fu organs to tonify Qi, regulate the spleen and stomach, and unblock the intestines, offering a more direct and concentrated therapeutic effect on gastrointestinal dysfunction than distal acupoints on the limbs (Z. B. 1999).

From a modern medical perspective, the theoretical basis of AA aligns closely with the concept of the “brain–gut axis” and the enteric nervous system (ENS), often referred to as the “second brain” (Liu et al. 2023). PSC is primarily caused by central nervous system lesions that disrupt the autonomic regulation of the gastrointestinal tract. Because the abdominal wall shares overlapping segmental innervation with the internal organs, the localized, shallow stimulation of AA can directly modulate the ENS. This stimulation enhances parasympathetic nerve excitability, promotes gastrointestinal motility, and regulates the release of key neurotransmitters (such as 5‐HT and VIP) more efficiently than general somatic acupuncture (Yu 2020). Furthermore, AA is characterized by shallow needle insertion, making it virtually painless. This is particularly advantageous for stroke patients, who frequently present with hemiplegia, spasticity, or sensory deficits in their extremities, making traditional deep‐insertion body acupuncture less tolerable or harder to implement.

Acupuncture improves constipation and gastrointestinal dysfunction after stroke through multiple mechanisms. First, acupuncture can affect autonomic nervous system balance and promote the coordinated actions of the sympathetic and parasympathetic nerves, thereby enhancing intestinal peristalsis, shortening defecation time, and improving the brain regulation of intestinal reflexes to restore normal gastrointestinal motility (Wu et al. 2023). Second, acupuncture regulates the secretion of gastrointestinal hormones (such as gastrin, serotonin, and vasoactive intestinal peptide), which normalizes the levels of these hormones in patients or animal models, thereby promoting intestinal peristalsis and the defecation reflex (Leng et al. 2019). In addition, acupuncture may exert an anti‐inflammatory effect by regulating the levels of inflammatory factors and enhancing local blood circulation, resulting in nerve cell damage reduction and intestinal function improvement (Liu and Hu 2024). Meanwhile, studies suggest that acupuncture may regulate the structure of the intestinal flora, improve dysbiosis, and thus restore intestinal microecological balance. Finally, acupuncture may promote nerve repair and regeneration by upregulating the expression of neurotrophic factors (such as nerve growth factor and brain‐derived neurotrophic factor), thereby providing theoretical support for neurological rehabilitation (Wu et al. 2023).

Our meta‐analysis showed that both MAA and AEA, in combination with CT, showed significantly improved therapeutic effects in PSC. MAA + CT and AEA + CT were more effective than CT alone, with RRs of 1.26 and 1.28, respectively. This supports the hypothesis that AA enhances the effects of CT by directly regulating gastrointestinal function through specific abdominal acupoints.

The significantly shorter time to first BM in the MAA + CT and AEA + CT groups further confirmed the potential of AA to promote bowel motility and accelerate constipation relief. These results were consistent with the theoretical mechanisms and clinical findings of AA.

However, the evidence regarding stool shape was inconclusive, with inconsistent findings and high heterogeneity. These findings highlight the current lack of evidence in the literature, thereby indicating the need for further studies exploring the specific effects of AA on stool consistency. Small sample sizes and the limited number of studies focusing on stool shape were the main factors contributing to this uncertainty.

Although primary outcomes (efficacy and time to first BM) enhance the use of AA for PSC, the quality of evidence is limited. The overall level of evidence was downgraded owing to the moderate‐to‐high risk of bias in many studies, small sample sizes, and significant heterogeneity, especially for secondary outcomes, such as stool shape. Furthermore, the variability in acupuncture protocols (including the duration, frequency, and type of AA) makes it difficult to draw definitive conclusions regarding their efficacy.

### 4.1 Strengths

Our study is the first meta‐analysis to isolate and specifically evaluate AA (including MAA and AEA) for PSC.

The distinct theoretical and clinical characteristics of AA underscore the novelty and necessity of this meta‐analysis. We acknowledge that three previous meta‐analyses have evaluated the efficacy of acupuncture for constipation (Sun et al. 2023; Han et al. 2021; Song et al. 2024). However, those studies evaluated “general acupuncture” as a broad category, pooling together various modalities, such as scalp, auricular, and traditional body acupuncture. This “lumping” approach inevitably introduces substantial clinical heterogeneity and obscures the specific effect sizes of individual acupuncture systems.

By narrowing our focus to this highly standardized and localized intervention, we significantly reduced the clinical heterogeneity associated with different acupuncture modalities. This is evidenced by the minimal statistical heterogeneity observed in our primary efficacy outcomes. Furthermore, by integrating TSA and GRADE assessments, our study provides a more rigorous and nuanced evaluation of the evidence than previous broad reviews. Therefore, this study not only confirms the general benefits of acupuncture but specifically validates AA as a highly targeted, safe, and effective non‐pharmacological intervention for PSC, providing more precise and actionable evidence for clinical decision‐making.

### 4.2 Limitations of Included Studies

Despite the encouraging results of this meta‐analysis, several limitations should be acknowledged. First, the meta‐analysis included only 12 RCTs with a relatively small total sample size (943 participants). This limited number of studies, particularly for secondary outcomes, such as stool shape, reduces statistical precision and may introduce small‐study bias to the results. Second, the methodological quality of the included studies poses a challenge. Ten studies had a moderate risk of bias, and two had a high risk of bias. The reliability of the results may have been compromised by issues, such as missing outcome data, lack of blinding, and inconsistent outcome measurement methods. These biases are particularly critical when interpreting secondary outcomes, which varied significantly between studies. Third, substantial heterogeneity was observed across multiple outcome measures, including stool shape (*I* ^2^ = 81%) and time to first BM for AEA + CT (*I* ^2^ = 90%). This variation likely stems from differences in acupuncture protocols (e.g., acupuncture type, frequency, and duration), making it difficult to establish a standardized treatment approach. Such heterogeneity suggests that the included studies may not be directly comparable, thereby reducing the overall quality of the evidence. Fourth, a specific methodological limitation concerns the definition of the primary outcome, “total response rate” or “efficacy.” In the included studies, this composite outcome was generally defined on the basis of improvements in defecation frequency, difficulty, and accompanying symptoms. However, the specific criteria and thresholds for “effective” versus “ineffective” varied slightly across trials and were not fully standardized according to international guidelines (e.g., Rome IV criteria). Although this reliance on study‐specific definitions is common in acupuncture research conducted in China, it introduces a degree of clinical heterogeneity that may affect the comparability of results. Consequently, the pooled “total response rate” should be interpreted as a broad indicator of clinical improvement rather than a precise, standardized metric. This variation underscores the need for future trials to adopt internationally recognized, validated outcome measures—such as the complete spontaneous BM (CSBM) rate or standardized quality of life scores—to enhance the precision and global relevance of the findings. Fifth, a critical limitation that moderately impacts the interpretation of our findings is the restricted external validity and generalizability. It must be explicitly emphasized that all 12 RCTs were conducted exclusively in China. Consequently, the study populations share similar genetic backgrounds, dietary habits, and gut microbiome profiles—factors intrinsically linked to gastrointestinal motility. Furthermore, the Chinese healthcare system seamlessly integrates TCM with conventional Western medicine. This means that the “CT” baseline, patient compliance, and cultural acceptance of acupuncture might differ significantly from standard poststroke care protocols in other countries. Because of these geographical and cultural constraints, it remains uncertain whether the observed efficacy can be directly extrapolated to other global populations and healthcare systems. Sixth, regarding treatment safety, although some included trials mentioned the recording of adverse events, none actually reported any occurrences. This complete absence of reported adverse events should be interpreted with caution. It is highly probable that this reflects a methodological limitation—specifically, the underreporting or inadequate monitoring of mild, transient adverse events (such as minor subcutaneous hematoma or localized pain at the needle site)—rather than a true absolute absence of side effects. This potential for underreporting limits our ability to provide a fully comprehensive and balanced assessment of the safety profile of AA for PSC. Future trials must implement standardized, rigorous protocols for the monitoring and reporting of all adverse events to ensure transparency. Finally, these combined factors affect the certainty of the evidence. In the GRADE assessment, the overall quality of evidence was downgraded to low or very low owing to the risk of bias, small sample sizes, and significant heterogeneity. Moreover, the TSA indicated that the cumulative evidence remains inconclusive, as the RIS threshold was not reached. In conclusion, although AA shows promise as an intervention for PSC within its specific context, our conclusions should be interpreted with caution globally. To establish AA as a universally applicable, evidence‐based therapy, future high‐quality, large‐scale, and international multicenter RCTs are urgently needed. These trials should include diverse ethnic populations and operate under different healthcare frameworks to truly validate the external applicability and comparative efficacy of this therapy.

## 5 Conclusion

This meta‐analysis provides preliminary evidence that combining MAA and AEA with CT may be effective in improving PSC. The results showed that both MAA + CT and AEA + CT significantly improved the efficacy of CT and shortened the time to first BM. These findings are consistent with the theoretical mechanisms of acupuncture, including gastrointestinal motility promotion, autonomic nervous system balance, and neurohormonal modulation, which may restore bowel function.

However, evidence regarding the effect of AA on stool shape is unclear, with significant variations between studies. Factors, such as the small number of studies, moderate‐to‐high risk of bias, and variations in acupuncture protocols, limit the strength of the conclusions drawn from this analysis. Furthermore, the overall quality of evidence was downgraded in the GRADE assessment, suggesting that more rigorous studies are needed to better assess the long‐term effects and optimize treatment protocols.

TSA further emphasized the uncertainty of cumulative evidence, indicating the need for more high‐quality trials to confirm the efficacy of AA for PSC.

Therefore, although AA is a promising adjunct treatment for PSC, further well‐designed, large‐scale RCTs are needed to validate these findings, address the gaps identified in the literature, and determine the optimal acupuncture regimen for clinical practice.

## Author Contributions

**Wulam Rukye**: data curation, formal analysis, investigation, methodology, project administration, software, validation, visualization, writing – original draft, writing – review and editing. **Hongzhi Yang**: data curation, formal analysis, investigation, project administration, writing – original draft. **Yingjia Li**: data curation, formal analysis, software, writing – review and editing. **Xin Li**: data curation, formal analysis, software, validation, writing – review and editing. **Nenggui Xu**: conceptualization, funding acquisition, resources, supervision, writing – review and editing. **Zhennan Wu**: conceptualization, funding acquisition, resources, supervision, visualization, writing – review and editing.

## Funding

The authors have nothing to report.

## Ethics Statement

The authors have nothing to report.

## Conflicts of Interest

The authors declare no conflicts of interest.

## Supporting information




**Supplementary Tables**: brb371442‐sup‐0001‐TableS1‐S8.docx

**Supplementary Figure**: brb371442‐sup‐0002‐FigureS2.png

**Supplementary Figure**: brb371442‐sup‐0003‐FigureS3.png

## Data Availability

The data supporting the findings of this study are available within the article and/or its Supporting Information.
