# Supplementary material for: Efficacy of Abdominal Acupuncture in Poststroke Constipation: A Systematic Review and Meta‐Analysis With Trial Sequential Analysis
Source: Brain Behav. 2026 Apr 29;16(5):e71442. doi: 10.1002/brb3.71442 (PMC13128978; doi:10.1002/brb3.71442)
Supplement: Supplementary file 1 — Supplementary Tables: brb371442‐sup‐0001‐TableS1‐S8.docx [file BRB3-16-e71442-s003.docx]

Supplementary 1. search strategy

**Table 1:** PubMed Search

| **NO** | **Search Details** | **Results** |
| --- | --- | --- |
| #1 | "Acupuncture Therapy"[Mesh] | 30,710 |
| #2 | ((((((((Acupuncture Treatmen[Title]) OR (Acupuncture Treatments[Title])) OR (Therapy, Acupuncture[Title])) OR (Pharmacoacupuncture Treatment[Title])) OR (Treatment, Pharmacoacupuncture[Title]))OR(electroacupuncture[Title])) OR (traditional acupuncture[Title])) OR (fire acupuncture[Title])) | 5,836 |
| #3 | "Stroke"[Mesh] | 184,558 |
| #4 | (((((((((((((((((((((Strokes) OR (Cerebrovascular Accident)) OR (Cerebrovascular Accidents)) OR (Cerebral Stroke)) OR (Cerebral Strokes)) OR (Strokes, Cerebral)) OR (Cerebrovascular Apoplexy)) OR (Apoplexy, Cerebrovascular)) OR (Vascular Accident, Brain)) OR (Brain Vascular Accident)) OR (Brain Vascular Accidents)) OR (Vascular Accidents, Brain)) OR (Cerebrovascular Stroke)) OR (Cerebrovascular Strokes)) OR (Stroke, Cerebrovascular)) OR (Strokes, Cerebrovascular)) OR (Apoplexy)) OR (Cerebrovascular Accident)) OR (Cerebrovascular Accident)) OR (Acute Stroke)) OR (Cerebrovascular Accident, Acute)) OR (Acute Cerebrovascular Accident) | 479,205 |
| #5 | "Constipation"[Mesh] | 16,794 |
| #6 | (Colonic Inertia) OR (Dyschezia) | 36,735 |
| #7 | "Randomized Controlled Trials as Topic"[Mesh] | 177,697 |
| #8 | ((Clinical Trials, Randomized) OR (Trials, Randomized Clinical)) OR (Controlled Clinical Trials, Randomized) | 791,926 |
| #9 | 1 OR 2 | 31,775 |
| #10 | 3 OR 4 | 479,205 |
| #11 | 5 OR 6 | 36,735 |
| #12 | 7 OR 8 | 791,926 |
| #13 | 9 AND 10 AND 11 AND 12 Filters:from 2000-2024 | 69 |

**Table 2:** Cochrane Library Search

| NO | Search Details | Results |
| --- | --- | --- |
| #1 | acupuncture | 22627 |
| #2 | (acupuncture treatment):ab,ti,kw OR (fire acupuncture):ab,ti,kw OR (warm acupuncture):ab,ti,kw OR (electro-acupuncture):ab,ti,kw | 14780 |
| #3 | Stroke | 89364 |
| #4 | ( Cerebrovascular Apoplexy):ab,ti,kw OR (rain Vascular Accident):ab,ti,kw OR (Brain Vascular Accidents):ab,ti,kw OR (Cerebrovascular Stroke):ab,ti,kw OR (Cerebrovascular Strokes):ab,ti,kw OR ( Cerebral Stroke):ab,ti,kw OR (Cerebral Strokes):ab,ti,kw OR (Brain Infarction):ab,ti,kw OR (Angst):ab,ti,kw | 28254 |
| #5 | constipation | 16992 |
| #6 | ( dyschezia):ab,ti,kw OR (constipation):ab,ti,kw OR (constipating):ab,ti,kw | 16267 |
| #7 | #1OR#2 | 22627 |
| #8 | (trial):ab,ti,kw OR (clinical trials as topic):ab,ti,kw OR (random):ab,ti,kw | 1176242 |
| #9 | #3OR#4 | 91360 |
| #10 | #5OR#6 | 17074 |
| #11 | 7 AND 8 AND 9 AND 10 | 26 |
|  | (2000-2024) |  |

#### **Table 3:** Embase Search

| **NO** | **Search Details** | **Results** |
| --- | --- | --- |
| #1 | 'acupuncture' OR 'acupuncture'/exp OR acupuncture OR 'pharmacopuncture':ab OR 'acupuncture treatment':ab OR 'acupuncture treatments':ab OR 'treatment, acupuncture':ab OR 'therapy, acupuncture':ab OR 'pharmacoacupuncture treatment':ab OR 'treatment, pharmacoacupuncture':ab OR 'pharmacoacupuncture therapy':ab OR 'therapy, pharmacoacupuncture':ab OR 'acupotomy':ab OR 'acupotomies':ab OR 'acupunctures, ear':ab OR 'ear acupunctures':ab OR 'auricular acupuncture':ab OR 'ear acupuncture':ab OR 'acupuncture, auricular':ab OR 'auricular acupunctures':ab OR 'acupunctures, auricular':ab OR 'acupuncture point':ab OR 'point, acupuncture':ab OR 'points, acupuncture':ab OR 'acupoints':ab OR 'acupoint':ab OR 'moxabustion':ab | 73847 |
| #2 | strok OR 'strokes':ab OR 'cerebrovascular accident':ab OR 'cerebrovascular accidents':ab OR 'cva (cerebrovascular accident)':ab OR 'cvas (cerebrovascular accident)':ab OR 'apoplexy, cerebrovascular':ab OR 'cerebrovascular apoplexy':ab OR 'vascular accident, brain':ab OR 'brain vascular accident':ab OR 'vascular accidents, brain':ab OR 'brain vascular accidents':ab OR 'cerebrovascular stroke':ab OR 'strokes, cerebrovascular':ab OR 'stroke, cerebrovascular':ab OR 'cerebrovascular strokes':ab OR 'apoplexy':ab OR 'cerebral stroke':ab OR 'cerebral strokes':ab OR 'stroke, cerebral':ab OR 'strokes, cerebral':ab OR 'stroke, acute':ab OR 'acute stroke':ab OR 'acute strokes':ab OR 'strokes, acute':ab OR 'cerebrovascular accident, acute':ab OR 'acute cerebrovascular accident':ab OR 'cerebrovascular accidents, acute':ab OR 'acute cerebrovascular accidents':ab | 242354 |
| #3 | constipation OR 'colonic inertia':ab OR 'dyschezia':ab OR 'constipation':ab OR 'constipated':ab OR 'constipating':ab | 131884 |
| #4 | 'trial':ab OR 'clinical trials as topic':ab OR 'clinical trial':ab OR 'random*':ab OR 'random allocation':ab OR 'therapeutic use':ab | 2573807 |
| #5 | 1 AND 2 AND 3 AND 4 | 10 |

**Table 4 :** Web of Science Search

| **NO** | **Search Details** | **Results** |
| --- | --- | --- |
| #1 | ((((TS=(constipation)) OR TS=(colonic inertia)) OR TS=(constipated)) OR TS=(constipating)) | 50824 |
| #2 | ((((((((((((((((((((((((((((TS=(Acupuncture)) OR TS=(Acupuncture Therapy)) OR TS=(Acupuncture, Ear)) OR TS=(Acupuncture Points)) OR TS=(Moxibustion)) OR TS=(Pharmacopuncture)) OR TS=(Acupuncture Treatment)) OR TS=(Acupuncture Treatments)) OR TS=(Treatment, Acupuncture)) OR TS=(Therapy, Acupuncture)) OR TS=(pharmacoacupunture Treatment)) OR TS=(Treatment, pharmacoacupunture)) OR TS=(pharmacoacupunture Therapy)) OR TS=(Therapy, pharmacoacupunture)) OR TS=(autotomy)) OR TS=(autotomies)) OR TS=(acupuncture, Ear)) OR TS=(Ear acupuncture)) OR TS=(Auricular Acupuncture)) OR TS=(Ear Acupuncture)) OR TS=(Acupuncture, Auricular)) OR TS=(acupuncture, Auricular)) OR TS=(Auricular acupuncture)) OR TS=(Acupuncture Point)) OR TS=(Point, Acupuncture)) OR TS=(Points, Acupuncture)) OR TS=(Acupoints)) OR TS=(Acupoint)) OR TS=(moxibustion) and Preprint Citation Index (Exclude – Database) | 54108 |
| #3 | (((((((((((((((((((((((((((TS=(Strokes)) OR TS=(Cerebrovascular Accident)) OR TS=(Cerebrovascular Accidents)) OR TS=(CVA (Cerebrovascular Accident))) OR TS=(cmas (Cerebrovascular Accident))) OR TS=(Cerebrovascular Apoplexy)) OR TS=(Apoplexy, Cerebrovascular)) OR TS=(Vascular Accident, Brain)) OR TS=(Brain Vascular Accident)) OR TS=(Brain Vascular Accidents)) OR TS=(Vascular Accidents, Brain)) OR TS=(Cerebrovascular Stroke)) OR TS=(Cerebrovascular Strokes)) OR TS=(Stroke, Cerebrovascular)) OR TS=(Strokes, Cerebrovascular)) OR TS=(Apoplexy)) OR TS=(Cerebral Stroke)) OR TS=(Cerebral Strokes)) OR TS=(Stroke, Cerebral)) OR TS=(Strokes, Cerebral)) OR TS=(Stroke, Acute)) OR TS=(Acute Stroke)) OR TS=(Acute Strokes)) OR TS=(Strokes, Acute)) OR TS=(Cerebrovascular Accident, Acute)) OR TS=(Acute Cerebrovascular Accident)) OR TS=(Acute Cerebrovascular Accidents)) OR TS=(Cerebrovascular Accidents, Acute) and Preprint Citation Index (Exclude – Database) | 637732 |
| #4 | ((((((TS=(clinical)) AND TS=(trial)) OR TS=(clinical trials as topic)) OR TS=(clinical trial)) OR TS=(random*)) OR TS=(random allocation)) OR TS=(therapeutic use) | 7217020 |
| #5 | #1 AND #2 AND #3 AND #4 | 23 |
| #6 | #5and Preprint Citation Index (Exclude – Database) and 2000-2024 (Publication Years) | 23 |

**Table 5:** CBM Search

| NO | Search details | results |
| --- | --- | --- |
| #1 | ("针刺"[标题:智能] OR "针刺疗法"[标题:智能] OR "温针疗法"[标题:智能] OR "传统针灸"[标题:智能] OR "电针"[标题:智能]) OR "穴位"[常用字段:智能]OR "温针疗法"[不加权:扩展] OR "逆针灸"[不加权:扩展]OR "针刺"[不加权:扩展] OR "针刺, 耳"[不加权:扩展] OR "针刺泻法"[不加权:扩展] OR "针刺补法"[不加权:扩展] OR "针刺补泻"[不加权:扩展] OR "排针刺法"[不加权:扩展] OR "放血疗法"[不加权:扩展] OR "微针疗法"[不加权:扩展] OR "火针疗法"[不加权:扩展] OR "电针疗法"[不加权:扩展] OR "电针"[不加权:扩展] 2000-2024[日期] | 93215 |
| #2 | "中风"[标题:智能] OR "脑卒中"[标题:智能] OR "卒中"[标题:智能] OR "脑血管意外"[标题:智能] OR "脑梗死"[标题:智能] OR "脑出血"[常用字段:智能] OR "颅内出血"[常用字段:智能] OR "脑梗死"[常用字段:智能] OR "中风"[常用字段:智能] OR "卒中"[常用字段:智能] OR "缺血性卒中"[常用字段:智能] OR "出血性卒中"[常用字段:智能] OR "栓塞性卒中"[常用字段:智能] OR "颅内栓塞"[常用字段:智能] OR "颅内栓塞和血栓形成"[常用字段:智能] OR "蛛网膜下腔出血"[常用字段:智能] OR "脑梗塞"[常用字段:智能] OR "脑栓塞"[常用字段:智能] OR "脑血栓形成"[常用字段:智能] OR "脑溢血"[常用字段:智能] OR "脑血管意外"[常用字段:智能])) OR (("出血性卒中"[不加权:扩展] OR "栓塞性卒中"[不加权:扩展] OR "颅内栓塞"[不加权:扩展] OR "颅内栓塞和血栓形成"[不加权:扩展] OR "蛛网膜下腔出血"[不加权:扩展]) OR ("脑出血"[不加权:扩展] OR "颅内出血"[不加权:扩展] OR "脑梗死"[不加权:扩展] OR "中风"[不加权:扩展] OR "卒中"[不加权:扩展] OR "缺血性卒中"[不加权:扩展] | 397946 |
| #3 | "便秘"[标题:智能] OR "结肠惯性"[标题:智能] OR "大便困难"[标题:智能]OR "便干"[常用字段:智能] OR "大便困难"[常用字段:智能])) OR (("便秘"[不加权:扩展] OR "便秘"[不加权:扩展]) OR "排便异常"[不加权:扩展] | 35748 |
| #4 | "临床随机对照实验"[常用字段:智能] OR "随机对照实验"[常用字段:智能] OR "clinical"[常用字段:智能] OR "randomized"[常用字段:智能] AND "controlled"[常用字段:智能] AND "trial"[常用字段:智能] OR "randomized"[常用字段:智能] OR "experiment"[常用字段:智能] | 4986432 |
| #5 | 1 AND 2 AND 3 AND 4 | 134 |

**Table 6:** CNKI Search

| **NO** | **Search Details** | **Results** |
| --- | --- | --- |
| #1 | （主题：针刺） OR （主题：针刺疗法） OR（主题：针灸） OR （主题：针灸疗法） OR （主题：温针疗法） OR （主题：火针疗法） OR （主题：电针） OR（主题：耳针） OR （主题：头针） | 253124 |
| #2 | （主题：便秘） OR （主题：大便秘结） OR （主题：大便不利） | 48610 |
| #3 | （摘要：随机对照试验（精确）） OR （摘要：临床试验（精确）） OR（摘要：RCT（精确）） | 110705 |
| #4 | （主题：针刺） OR （主题：针刺疗法） OR （主题；针灸） OR（主题：针负疗法） OR （主题：温针疗法）OR（生题：火针疗法） OR （主题：电针）OR （主题：耳针） OR （主题：头针） AN D （主题：卒中） OR （主题：中风） OR （主题：脑卒中）OR （主题：脑中风）OR （主题：脑血管意外）OR （主题：脑梗死）发表时间：2000-01-02到2024-11-20 | 27560 |
| #5 | 2 AND 3 AND 4 | 280 |

**Table 7:** Wanfang Search

| NO | Search Details | **Results** |
| --- | --- | --- |
| #1 | (主题:("针刺") or 主题:("针刺疗法") or 主题:("针灸") or 主题:(针灸疗法) or 主题:(针刺穴位) or 主题:(电针)OR主题: （电针) OR主题: （穴位"）) and 发表时间:2000-2024 | 330633 |
| #2 | (主题:("中风") or 主题:("卒中") or 主题:("脑卒中") or 主题:(脑中风) or 主题:(脑血管意外)) and 发表时间:2000-2024 | 539203 |
| #3 | (主题:("便秘") or 主题:("大便秘结") or 主题:("大便不利") or 主题:(大便困难) or 主题:(结肠惯性)OR主题: "颅内栓塞"OR "主题:颅内栓塞和血栓形成"OR 主题:"蛛网膜下腔出血" OR主题: "脑梗塞"OR主题: "脑栓塞" OR 主题:"脑血栓形成"[常用字段:智能] OR主题: "脑溢血"）) and 发表时间:2000-2024 | 102249 |
| #4 | (摘要:(随机对照试验) or 摘要:(临床随机对照) or 摘要:(临床试验) or 摘要:(RCT)) and 发表时间:2000-2024 | 1747487 |
| #5 | ((主题:("便秘" OR "排便" OR "便干" OR "大便困难") and 主题:("卒中" OR "中风" OR "脑出血" OR "脑梗死" OR "脑血管意外" OR "脑梗" OR "脑缺血" OR "脑梗塞" OR "脑血栓" OR "脑栓塞" OR "缺血性脑卒中" OR "缺血性中风" OR "蛛网膜下腔出血" OR "脑溢血")) or 主题:("卒中后便秘" OR "中风后便秘")) and 主题:("针灸" OR "针刺" OR "温针灸" OR "火针" OR "电针" OR "针" OR "刺" OR "毫针" OR "刺血" OR "头针" OR "体针" OR "腹针" OR "指针" OR "穴位" OR "传统医疗") and 摘要:("随机对照试验" OR "随机对照研究" OR "RCT" OR "随机" OR "对照" OR "控制组" OR "安慰剂" OR "试验" OR "分组" OR "临床" OR "临床研究" OR "临床疗效")AND发表时间:2000-2024 | 301 |

**Table 8:** cqvip Search

| **NO** | **Search Details** | **Results** |
| --- | --- | --- |
| #1 | (((((题名或关键词=中风 OR 题名或关键词=脑卒中) OR 题名或关键词=stroke) AND (((((摘要=随机对照试验 OR 摘要=randomized clinical trials) OR 摘要=randomized controlled clinical trial) OR 摘要=randomized controlled trial) OR 摘要=randomized controlled trials) OR 摘要=randomized experiment) OR 摘要=rct) OR 摘要=随机对照实验) OR 摘要=随机对照研究))AND (years:[2000 TO 2024]) | 46 |
| #2 | (((((题名或关键词=中风 OR 题名或关键词=脑卒中) OR 题名或关键词=stroke) AND ((题名或关键词=针灸治疗 OR 题名或关键词=acupuncture) OR 题名或关键词=针刺治疗)) AND ((((((((摘要=随机对照试验 OR 摘要=randomized clinical trials) OR 摘要=randomized controlled clinical trial) OR 摘要=randomized controlled trial) OR 摘要=randomized controlled trials) OR 摘要=randomized experiment) OR 摘要=rct) OR 摘要=随机对照实验) OR 摘要=随机对照研究)) AND (years:[2000 TO 2024]) | 27 |
| #3 | ((((((题名或关键词=中风 OR 题名或关键词=脑卒中) OR 题名或关键词=stroke) AND ((题名或关键词=针灸治疗 OR 题名或关键词=acupuncture) OR 题名或关键词=针刺治疗)) AND ((((((摘要=随机对照试验 OR 摘要=randomized clinical trials) OR 摘要=randomized controlled clinical trial) OR 摘要=randomized controlled trial) OR 摘要=randomized controlled trials) OR 摘要=randomized experiment) OR 摘要=rct) AND (((题名或关键词=便秘 OR 题名或关键词=constipation) OR 题名或关键词=bound) OR 题名或关键词=大便不畅)) AND (years:[2000TO 2024]) | 0 |
| #4 | #1 AND #2 AND #3 | 0 |
