# Supplementary figures and images for: Efficacy of Abdominal Acupuncture in Poststroke Constipation: A Systematic Review and Meta‐Analysis With Trial Sequential Analysis
Source: Brain Behav. 2026 Apr 29;16(5):e71442. doi: 10.1002/brb3.71442 (PMC13128978; doi:10.1002/brb3.71442)

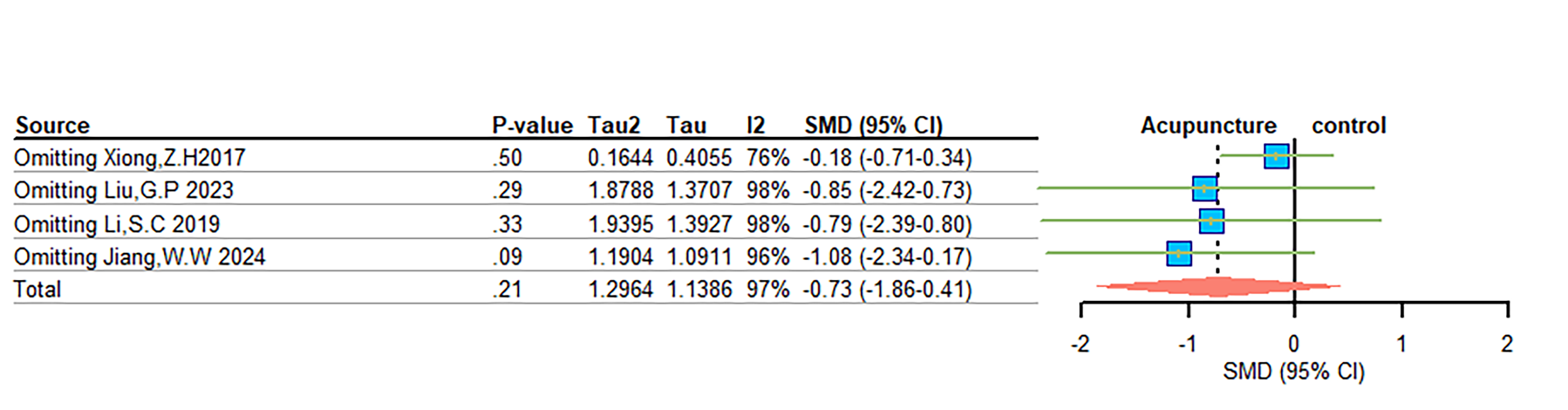

Supplement: Supplementary file 2 — Supplementary Figure: brb371442‐sup‐0002‐FigureS2.png [file BRB3-16-e71442-s002.png]

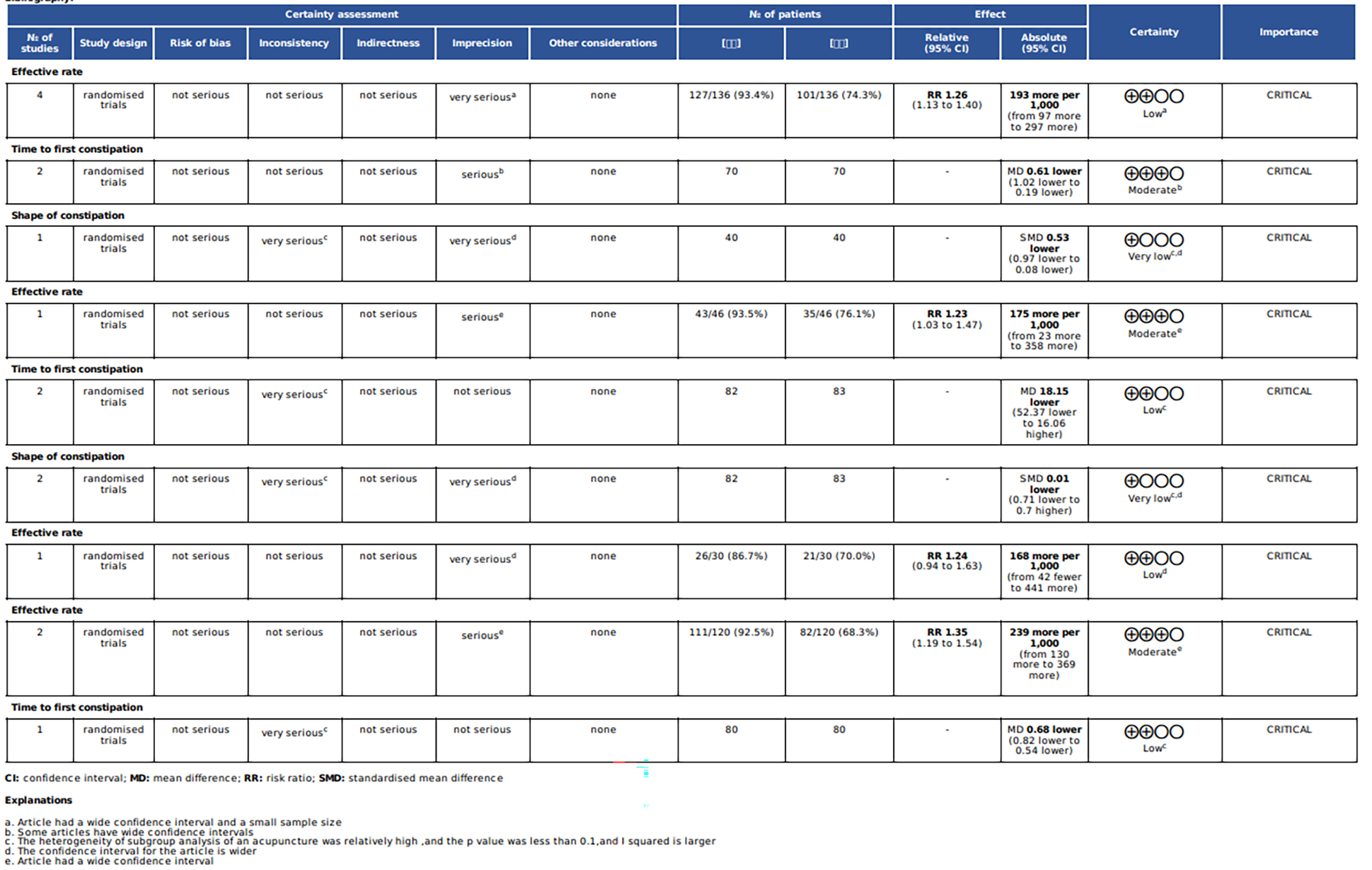

Supplement: Supplementary file 3 — Supplementary Figure: brb371442‐sup‐0003‐FigureS3.png [file BRB3-16-e71442-s001.png]
